# Role of Magnetic Resonance Imaging in the Evaluation of Trigeminal Neuralgia Using Steady-State Imaging

**DOI:** 10.7759/cureus.60071

**Published:** 2024-05-10

**Authors:** Nikita Bora, Pratapsingh Parihar, Nishant Raj, Bhagyasri Nunna, Neha D Shetty

**Affiliations:** 1 Radiodiagnosis, Datta Meghe Institute of Higher Education and Research, Wardha, IND

**Keywords:** constructive interference in steady-state (ciss), fast imaging employing steady-state acquisition (fiesta), mri, magnetic resonance imaging, trigeminal neuralgia

## Abstract

Trigeminal neuralgia (TN) poses diagnostic challenges due to its complex origins, often associated with neurovascular compression. Advanced imaging techniques, particularly magnetic resonance imaging (MRI) with the fast imaging employing steady-state acquisition (FIESTA) sequence, offer crucial insights into TN pathophysiology. This prospective cross-sectional observational study aimed to elucidate MRI's utility in diagnosing TN and correlating imaging findings with clinical manifestations and treatment outcomes. A cohort of 41 patients clinically suspected of TN underwent MRI evaluation at Acharya Vinoba Bhave Rural Hospital, Sawangi (Meghe), Wardha, utilizing various sequences including FIESTA. Analysis revealed a higher incidence among females, predominant unilateral presentation, and a higher prevalence of abnormal MRI findings, with neurovascular compression as the leading etiology. Correlation analysis demonstrated significant associations between facial pain localized to the trigeminal nerve distribution, triggering factors, and abnormal MRI findings. Gender distribution did not significantly influence MRI findings. Treatment outcomes favored microvascular surgery over conservative management in cases of neurovascular compression. This study underscores MRI's pivotal role, particularly FIESTA, in TN evaluation, guiding personalized treatment strategies and emphasizing the importance of integrated clinical and imaging approaches. Further research is warranted to validate these findings and explore additional imaging modalities for a deeper understanding of TN pathogenesis.

## Introduction

Trigeminal neuralgia presents as intense intermittent pain akin to electric shocks affecting areas innervated by the trigeminal nerve. This pain, typically unilateral and sudden in onset, recurs frequently throughout the day. The trigeminal nerve, a mixed cranial nerve comprising sensory and motor components, contains a motor nucleus responsible for jaw muscle control situated in the pons. Its sensory nuclei, including the mesencephalic, primary sensory, and spinal nuclei, handle various sensory functions such as proprioception and tactile sensation across the face and jaw. The cisternal segment of the trigeminal nerve, originating from the pons, primarily houses the sensory portion and a few minor motor roots. This segment is particularly susceptible to neurovascular conflicts, notably at the root entry zone where central-to-peripheral myelin conversion occurs [1-3]. 

The trigeminal nerve's course extends through the cisternal space, reaching Meckel's cave via the porus trigeminus, where it forms the gasserian or trigeminal ganglion. From this ganglion, the three divisions of the trigeminal nerve-ophthalmic (V1), maxillary (V2), and mandibular (V3) emerge, each providing sensory innervation to distinct facial regions. Trigeminal neuralgia primarily affects women, predominantly on the right side of the face, typically manifesting between the fifth and eighth decades of life, with higher prevalence in rural areas [4-6]. 

Neurovascular contact in the prepontine cistern, particularly involving the superior cerebellar artery and its branches, stands as the most common cause of trigeminal neuralgia. Other potential causes vary depending on the site of involvement, ranging from compression by tumors or aneurysms in the prepontine cistern to brainstem lesions like gliomas, infarctions, multiple sclerosis, or cavernomas. MRI, particularly utilizing sequences like 3D-FIESTA (fast imaging employing steady-state acquisition), serves as the primary imaging tool for diagnosing trigeminal neuralgia, though challenges like artifacts from natural fluid flow necessitate techniques like steady-state free precession (SSFP) sequences for better image quality [7,8]. 

Microvascular decompression (MVD) has emerged as the primary treatment for trigeminal neuralgia, targeting neurovascular compression. Imaging plays a crucial role in surgical planning, aiding surgeons in identifying the compressing vessel, its nature (artery or vein), and the affected segment of the trigeminal nerve, thereby influencing post-MVD prognosis [8-10]. 

In this discourse, we embark on a journey elucidating the pivotal role of MRI, with a particular spotlight on the utility of the FIESTA or constructive interference in steady-state (CISS) sequence, in comprehensively evaluating TN. Our exploration traverses various dimensions of TN, encompassing gender predilection, branch distribution, laterality, clinical manifestations, and underlying etiologies as unveiled through MRI diagnostics. Moreover, we delve into the intricate interplay between arterial structures, grading of neurovascular compression, and the nexus between clinical symptomatology and imaging revelations.

## Materials and methods

Aim

The aim of this study was to identify the potential causes of pain in clinically suspected patients of trigeminal neuralgia by using magnetic resonance imaging.

Objectives

This study sets out to investigate several objectives concerning trigeminal neuralgia and its diagnostic and therapeutic aspects. Firstly, it aims to scrutinize the imaging characteristics of the trigeminal nerve and its spatial relationship with adjacent anatomical structures. Secondly, it seeks to investigate the diverse etiologies contributing to pain in patients clinically suspected of trigeminal neuralgia. Thirdly, the study aims to delineate imaging findings in correlation with the clinical history of patients experiencing pain within the distribution area of the trigeminal nerve. Lastly, it aims to assess the response to treatment, whether medical intervention or microvascular surgery, if advised and pursued.

Methodology

This study utilized an MRI Machine, specifically the GE Brivo MR 355 model with 1.5 Tesla (GE HealthCare, Chandigarh, India), situated in the Department of Radiodiagnosis at Acharya Vinoba Bhave Rural Hospital, Sawangi (Meghe), Wardha. It employed a prospective cross-sectional observational design, targeting individuals seeking evaluation at the MRI department due to clinical suspicion of trigeminal neuralgia.

Sampling involved directing patients clinically suspected of trigeminal neuralgia to the Department of Radiodiagnosis at Acharya Vinoba Bhave Rural Hospital, Sawangi (Meghe), Wardha. The sample size of 41 patients was determined using Daniel's formula, with parameters set for a Type I error at 5%, Type II error at 20%, an estimated proportion at 88%, and an estimation error of 10%.

Inclusion criteria comprised individuals clinically suspected of trigeminal neuralgia, while exclusion criteria included patients under 18 years old, those with a history of previous trigeminal neuralgia surgery, patients with psychiatric disorders, individuals experiencing claustrophobia, those with metallic implants or cardiac pacemakers, and individuals who declined participation.

The procedural steps involved communicating details to patients and obtaining written informed consent. The MRI protocol encompassed various sequences: T1-weighted imaging (T1WI) in axial and sagittal sections, T2-weighted imaging (T2WI) in axial, coronal, and sagittal sections, axial and coronal fluid-attenuated inversion recovery (FLAIR), axial diffusion-weighted imaging (DWI), axial gradient recalled echo (GRE), 3D-FIESTA sequences, with parameters including repetition time of 4.8 ms, echo time of 1.4 ms, slice thickness of 1 mm, adjusted field of view (FOV) according to head size, matrix of 352 x 192, and number of excitations (NEX) of 3. Contrast-enhanced study with gadolinium-based contrast agents was administered when necessary.

## Results

**Figure 1:**
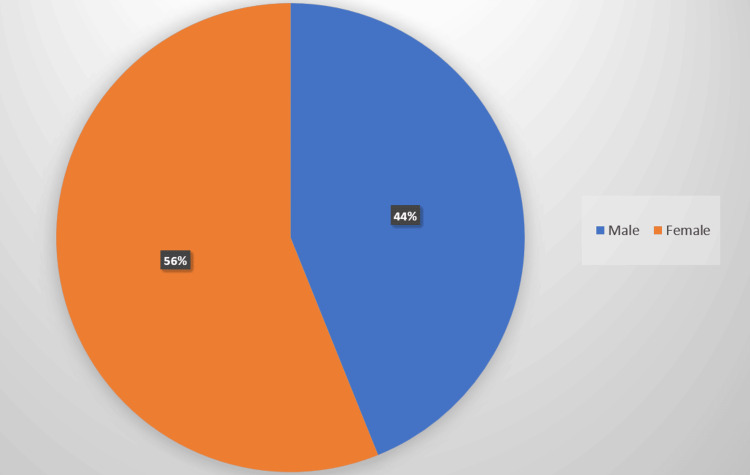
Gender distribution of the study participants

The study encompassed a cohort of 41 patients over a span of one year, all of whom underwent MRI imaging with the addition of a dedicated FIESTA sequence. Among the participants, 23 were female (Figure 1), constituting 56% of the sample, while 18 were male, making up 44%.

Analysis of age distribution (Table 1) revealed that the majority of patients (39%) belonged to the age group of 51-60 years, followed by 29% in the age bracket of 41-50 years. Additionally, 12% were aged over 70 years, with 9.7% each in the age groups of 18-30 years and 31-40 years.

**Table 1:** Age distribution of the study participants

Age Group	Number of Patients
18-30 years	4 (9.7%)
31 to 40 years	4 (9.7%)
41 to 50 years	12 (29%)
51 to 60 years	16 (39%)
>70 years	5 (12%)
Total	41

Clinical manifestations varied among the patients, with facial pain distributed along the trigeminal nerve reported in 93.5% of the cases. However, 6.4% experienced facial pain not within the typical distribution of the trigeminal nerve. Other prevalent symptoms included headache (51.6%), giddiness (9.6%), and triggering factors observed in 83.8% of the cases. Unilateral presentation of trigeminal neuralgia was predominant, accounting for 94% of the cases, while bilateral involvement was noted in 6% of the patients.

Regarding the distribution among trigeminal nerve branches, the maxillary division (V2) was the most affected, representing 51.6% of the cases, followed by the mandibular division (V3) at 32.2%. Notably, involvement of both V2 and V3 was observed in 16.1% of the cases. MRI findings revealed abnormalities in 85% of the cases, whereas 15% exhibited normal MRI results.

**Figure 2:**
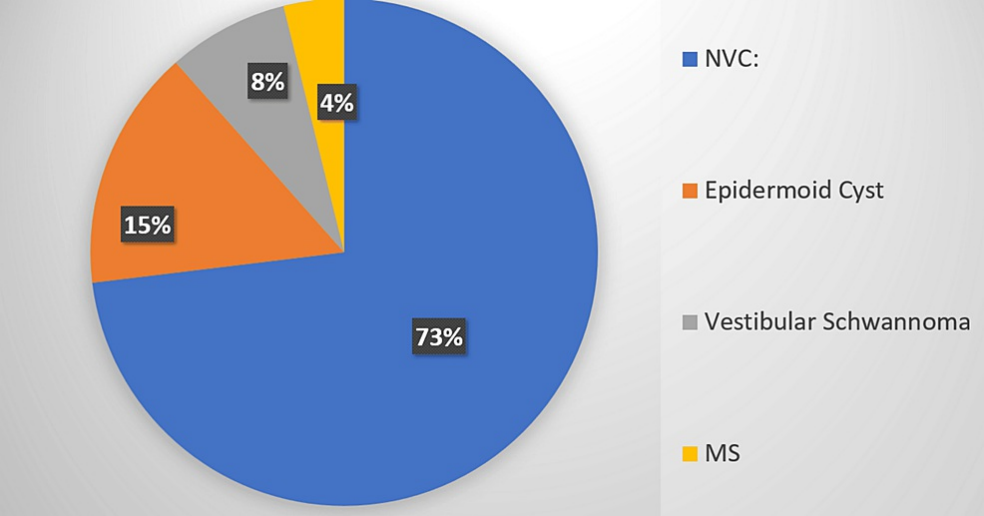
Etiology obtained on MRI NVC: Neurovascular compression MS: Multiple sclerosis

Analysis of the etiology (Figure 2) on MRI highlighted neurovascular compression as the leading cause, identified in 73% of the cases. Other notable etiologies included epidermoid cysts (15%), vestibular/trigeminal schwannomas (8%), and multiple sclerosis (4%). In terms of arterial involvement, the superior cerebellar artery (SCA) was implicated in 74% of the cases, followed by the anterior inferior cerebellar artery (AICA) in 26% of the cases.

​​​​​​

These findings underscore the complexity of trigeminal neuralgia pathology and emphasize the importance of detailed MRI evaluation, particularly utilizing the FIESTA sequence, in elucidating underlying mechanisms and guiding therapeutic strategies.

Correlation analysis

Correlation of Clinical Symptoms with Respect to MRI Findings (Table 2)

Facial pain was a prevalent symptom observed in 41 patients, among whom 39 experienced facial pain distributed along the trigeminal nerve. Strikingly, all 39 patients with facial pain in the distribution of the trigeminal nerve exhibited abnormal MRI findings, constituting 100% of the cases, whereas only 66.6% of those with facial pain in this distribution presented normal MRI results. The obtained p-value of 0.0063 indicated statistical significance, underscoring the association between facial pain localized to the trigeminal nerve distribution and abnormal MRI findings.

Among the study participants, 21 patients reported experiencing headache, while 20 did not. Of those with headache, 18 exhibited abnormal MRI findings, accounting for 51.4% of the cases, whereas three displayed normal MRI results. Conversely, among the 20 patients without headache, 17 presented abnormal MRI findings (48.5%) and three had normal MRI results (50%). Despite these observations, the obtained p-value of 0.32 indicated a lack of statistical significance in the association between headache and MRI findings.

Triggering factors were identified in 36 out of 41 cases, whereas five patients did not exhibit triggering factors. Among those with triggering factors, 94.2% presented abnormal MRI findings, with only three cases displaying normal MRI results. In contrast, among the five cases without triggering factors, 50% displayed normal MRI results, while 5.7% had abnormal findings. The obtained p-value of 0.003 signified statistical significance, highlighting the correlation between triggering factors and abnormal MRI findings.

Giddiness was reported in three patients, with two of them exhibiting abnormal MRI findings, constituting 5.7% of cases. Despite this observation, the obtained p-value of 0.37 indicated a lack of statistical significance in the association between giddiness and MRI findings. These findings underscore the varying degrees of association between clinical symptoms and MRI findings, emphasizing the importance of comprehensive evaluation in understanding the nuances of trigeminal neuralgia pathology.

**Table 2:** Correlation of clinical symptoms with MRI findings TN: Trigeminal neuralgia X^2^: Chi-square statistic

Symptoms		MRI FINDINGS				P-Value
		Abnormal(n=35)		Normal(n=6)		
		Count	%	Count	%	
Facial Pain	- (Not in TN Distribution)	0	0%	2	33.3%	χ2:7.2 P-Value:0.00713(Significant)
	+ (in TN distribution)	35	100%	4	66.6%	
Headache	-	17	48.5%	3	50%	χ2:0.0042 P-Value:0.94(Not Significant)
	+	18	51.4%	3	50%	
Triggering Factor	-	2	5.7%	3	50%	χ2:9.38 PValue:0.002(Significant)
	+	33	94.2%	3	50%	
Giddiness	-	33	94.2%	5	83.3%	χ2:0.906 P-value:0.34(Not Significant)
	+	2	5.7%	1	16.6%	

Correlation of Gender Distribution with MRI Findings (Table 3)

Among the total of 41 patients, 23 were female, and 18 were male. Of the 23 females, 22 exhibited abnormal MRI findings, while among the 14 males, 13 showed abnormal MRI findings. The resulting p-value was 0.8, indicating that these gender differences in MRI findings were not statistically significant.

**Table 3:** Correlation of MRI findings with gender distribution. X2: Chi square statistic

		MRI Diagnosis				P-Value
		Abnormal(n=35)		Normal(n=6)		
		Count	%	Count	%	
Sex	Male (n=18)	13	37.1%	5	83.3%	
	Female (n=23)	22	62.8%	1	16.6%	χ2:1.25 P-Value : 0.26(Not Significant)

Correlation of Laterality Distribution with MRI Findings

Regarding laterality distribution and MRI findings (Table 4), out of the 41 patients, unilateral cases predominated with 39 instances, while there were only two bilateral cases. Among the 39 unilateral cases, 34 had abnormal MRI findings, while five displayed normal MRI findings. Among the two cases with bilateral distribution, one exhibited abnormal MRI findings, while the other showed normal MRI findings.

**Table 4:** Correlation of MRI findings with laterality.

		MRI Diagnosis			
		Abnormal(n=35)		Normal(n=6)	
		Count	%	Count	%
Laterality	Bilateral (n=2)	1	2.85%	1	16.6%
	Unilateral (n=39)	34	97.14%	5	83.3%

Treatment Follow-up

Concerning treatment follow-up (Table 5), out of the 30 cases of neurovascular compression, 26 underwent microvascular surgery, while four were managed conservatively. Among the six patients with epidermoid cysts, five underwent surgery. three patients with vestibular schwannoma underwent surgery. One patient with multiple sclerosis was managed medically. One patient was lost to follow-up in our study.

**Table 5:** Treatment follow-up

ETIOLOGY	MODE OF TREATMENT	
Neurovascular Compression (n=30)	Microvascular Surgery	
	Medical Management	
Epidermoid Cyst (n=6)	Surgical Removal	
	Conservative Management	
Vestibular Schwannoma (n=3)	Surgery	
Multiple Sclerosis (n=1)	Medical Management	
Loss to follow-up (n=1)		

In the follow-up of patients with neurovascular compression (Table 6), 26 underwent microvascular surgery, while four were managed conservatively. Of the 26 who underwent microvascular surgery, 23 were completely symptom-free on follow-up, one had persistent symptoms, one experienced on-and-off complaints, and one exhibited deterioration in symptoms. Conversely, among patients managed conservatively with medical management, only one was completely symptom-free, one had persistent symptoms, one experienced on-and-off symptoms, and one exhibited deterioration in symptoms. The p-value obtained was 0.01, signifying statistical significance. This suggests a notable association between the type of treatment (microvascular surgery versus conservative management) and the observed outcomes during follow-up, with the difference in symptom outcomes likely not occurring by chance alone. Therefore, the type of treatment likely played a significant role in determining the symptom outcomes observed during follow-up.

**Table 6:** Results of treatment of neurovascular compression X2: Chi-square statistic

	Post-Microvascular Surgery (n=26)	Post-Medical Management (n=4)	P-value
Symptom-free	23	1	χ2:11.2 P-value:0.0007 (Significant)
Persistent symptoms	1	1	-
On and off complaints	1	1	
Deteoriation in symptoms	1	1	

## Discussion

Trigeminal neuralgia (TN) presents a formidable diagnostic puzzle owing to its intricate and multifaceted origins, often entwined with neurovascular compression as a central pathological mechanism. In recent years, advanced imaging methodologies have emerged as critical tools in unraveling the anatomical underpinnings of TN, furnishing invaluable perspectives into its pathophysiology and guiding therapeutic interventions. Among these imaging techniques, magnetic resonance imaging (MRI) has emerged as the cornerstone for TN evaluation, offering intricate visualization of craniofacial neurovascular structures with remarkable spatial resolution and tissue contrast [11-13].

In particular, the fast imaging employing steady-state acquisition (FIESTA) sequence, a specialized facet of MRI, has garnered considerable attention for its remarkable depiction of neurovascular structures, including the trigeminal nerve and neighboring blood vessels, with remarkable clarity. Leveraging steady-state free precession, FIESTA extends superior contrast between tissues and refined delineation of minute anatomical details, rendering it an indispensable tool in the comprehensive evaluation of TN. This advanced imaging modality empowers radiologists and clinicians to pinpoint neurovascular compression with precision, gauge the degree of nerve displacement or distortion, and assess the vascular contributions to TN pathology. Furthermore, FIESTA MRI facilitates the detection of secondary structural anomalies such as tumors or cysts impinging upon the trigeminal nerve, thereby enhancing diagnostic precision and guiding treatment decisions. Through its capacity to offer non-invasive, three-dimensional visualization of craniofacial neurovascular anatomy, FIESTA MRI has revolutionized the diagnostic paradigm of TN, furnishing clinicians with unparalleled insights into the underlying mechanisms driving this incapacitating condition [14,15].

Transitioning from the discourse on imaging techniques, our study findings shed light on the clinical and radiological facets of TN, further underscoring the pivotal role of MRI, particularly the FIESTA sequence, in its evaluation.

Demographic scrutiny of our study cohort, comprising 41 patients over a one-year span, unveiled a marginally higher representation of females (56%) in comparison to males (44%), consistent with extant literature indicating a higher incidence of TN among females. This gender asymmetry accentuates the significance of demographic variables in deciphering the epidemiology of TN and tailoring treatment modalities accordingly. Furthermore, the analysis of age distribution revealed a predominant representation of individuals aged 51-60 years, trailed by the 41-50 age bracket, suggesting a predilection for TN among middle-aged and elderly individuals. Such demographic insights into TN are pivotal for healthcare practitioners to discern and address the condition effectively.

Clinical presentations of TN exhibited variability among our patients, with the hallmark symptom of intense facial pain along the trigeminal nerve distribution reported in the majority of the cases (95.1%). Additionally, headache and triggering factors featured prominently among TN patients, underscoring the multifaceted clinical panorama of the condition. Crucially, unilateral presentation of TN predominated, consistent with its characteristic pattern, while bilateral involvement was noted in a smaller cohort of patients. This unilateral dominance underscores the significance of lateralization in both the diagnosis and management of TN, underscoring the importance of meticulous clinical assessment.

MRI findings in our study unveiled abnormalities in the majority of the cases (85.3%), with neurovascular compression emerging as the predominant etiology (73%). Other notable etiologies included epidermoid cysts, vestibular/trigeminal schwannomas, and multiple sclerosis, unraveling the diverse underlying mechanisms contributing to TN. Arterial compression, particularly by the superior cerebellar artery (SCA), was prevalent, further substantiating the vascular underpinnings of TN pathology. Moreover, grading of neurovascular conflict showcased varying degrees of compression, accentuating the intricacy of TN pathophysiology and the indispensability of detailed MRI assessment in guiding therapeutic interventions effectively.

Correlation analysis between clinical symptoms and MRI findings revealed significant associations between facial pain localized to the trigeminal nerve distribution, triggering factors, and abnormal MRI findings. Notably, all patients exhibiting facial pain within the trigeminal nerve distribution showcased abnormal MRI findings, reaffirming the utility of MRI in corroborating the anatomical basis of symptoms. Associations with headache and giddiness were less pronounced, suggesting potential multifactorial etiologies for these symptoms. Such correlation analysis underscores the importance of integrating clinical and imaging data for a comprehensive evaluation of TN.

Gender distribution analysis demonstrated no statistically significant differences in MRI findings between genders, indicating that TN pathology identified through imaging remains uninfluenced by gender. Unilateral presentation continued to dominate, with a higher incidence of abnormal MRI findings compared to bilateral cases, further underscoring the importance of lateralization in TN diagnosis. Treatment follow-up revealed more favorable outcomes with microvascular surgery compared to conservative management in patients with neurovascular compression, highlighting the efficacy of surgical intervention in alleviating TN symptoms.

In conclusion, our study underscores the indispensable role of MRI, particularly employing the FIESTA sequence, in the evaluation of TN patients. By amalgamating demographic characteristics, clinical manifestations, MRI findings, and treatment outcomes, we have gleaned invaluable insights into TN pathophysiology, which can inform personalized treatment strategies. Integrated clinical and imaging approaches are paramount for a comprehensive evaluation of TN and for optimizing therapeutic outcomes. Nevertheless, further research is warranted to validate these findings and explore additional imaging modalities for a deeper understanding of TN pathogenesis.

## Conclusions

In conclusion, our study underscores the indispensable role of MRI, particularly employing the FIESTA sequence, in the evaluation of TN patients. By amalgamating demographic characteristics, clinical manifestations, MRI findings, and treatment outcomes, we have gleaned invaluable insights into TN pathophysiology, which can inform personalized treatment strategies. Integrated clinical and imaging approaches are paramount for a comprehensive evaluation of TN and for optimizing therapeutic outcomes. Nevertheless, further research is warranted to validate these findings and explore additional imaging modalities for a deeper understanding of TN pathogenesis.

## References

[1] Rangaswamy VK, Srinivas MR, Basavalingu D, Nagaraj BR (2016). The role of magnetic resonance imaging in the evaluation of trigeminal neuralgia. Int J Anat Radiol Surg.

[2] Kikontzıalıs M, Koçak M (2017). Imaging evaluation of trigeminal neuralgia. J Istanb Univ Fac Dent.

[3] Katheriya G, Chaurasia A, Khan N, Iqbal J (2019). Prevalence of trigeminal neuralgia in Indian population visiting a higher dental care center in North India. Natl J Maxillofac Surg.

[4] Noble DJ, Scoffings D, Ajithkumar T, Williams MV, Jefferies SJ (2016). Fast imaging employing steady-state acquisition (FIESTA) MRI to investigate cerebrospinal fluid (CSF) within dural reflections of posterior fossa cranial nerves. Br J Radiol.

[5] Hughes MA, Frederickson AM, Branstetter BF, Zhu X, Sekula RF Jr (2016). MRI of the trigeminal nerve in patients with trigeminal neuralgia secondary to vascular compression. AJR Am J Roentgenol.

[6] Anwar H, Ramya Krishna M, Sadiq S, Ramesh Kumar R, Venkatarathnam V, Saikiran G (2022). A study to evaluate neurovascular conflict of trigeminal nerve in trigeminal neuralgia patients with the help of 1.5 T MR imaging. Egypt J Radiol Nucl Medi.

[7] Geneidi EA, Ali HI, Abdel Ghany WA, Nada MA (2016). Trigeminal pain: potential role of MRI. Egypt J Radiol Nucl Med.

[8] Love S, Coakham HB (2001). Trigeminal neuralgia: pathology and pathogenesis. Brain.

[9] Antonini G, Di Pasquale A, Cruccu G (2014). Magnetic resonance imaging contribution for diagnosing symptomatic neurovascular contact in classical trigeminal neuralgia: a blinded case-control study and meta-analysis. Pain.

[10] Vedaraju KS, Vijay SS, Madhusudana Y, Eada S (2021). Assessment of trigeminal neurovascular conﬂicts using 3D FIESTA-C sequence on a 3T MRI. Asian Journal of Medical Radiological Research.

[11] Chun-Cheng Q, Qing-Shi Z, Ji-Qing Z, Zhi-Gang W (2009). A single-blinded pilot study assessing neurovascular contact by using high-resolution MR imaging in patients with trigeminal neuralgia. Eur J Radiol.

[12] Lorenzoni J, David P, Levivier M (2012). Patterns of neurovascular compression in patients with classic trigeminal neuralgia: a high-resolution MRI-based study. Eur J Radiol.

[13] Miller JP, Acar F, Hamilton BE, Burchiel KJ (2009). Radiographic evaluation of trigeminal neurovascular compression in patients with and without trigeminal neuralgia. J Neurosurg.

[14] Peker S, Dinçer A, Necmettin Pamir M (2009). Vascular compression of the trigeminal nerve is a frequent finding in asymptomatic individuals: 3-T MR imaging of 200 trigeminal nerves using 3D CISS sequences. Acta Neurochir (Wien).

[15] Leal PR, Froment JC, Sindou M (2010). MRI sequences for detection of neurovascular conflicts in patients with trigeminal neuralgia and predictive value for characterization of the conflict (particularly degree of vascular compression) [in French]. Neurochirurgie.

